# Editorial: Personalized Sport and Exercise Nutrition

**DOI:** 10.3389/fnut.2019.00139

**Published:** 2019-08-28

**Authors:** Bryan Saunders, Ahmed El-Sohemy, Wim Derave

**Affiliations:** ^1^Applied Physiology and Nutrition Research Group, School of Physical Education and Sport, Rheumatology Division, Faculty of Medicine FMUSP, University of São Paulo, São Paulo, Brazil; ^2^Institute of Orthopaedics and Traumatology, Faculty of Medicine FMUSP, University of São Paulo, São Paulo, Brazil; ^3^Department of Nutritional Sciences, University of Toronto, Toronto, ON, Canada; ^4^Department of Movement and Sports Sciences, Ghent University, Ghent, Belgium

**Keywords:** hydration, supplementation, performance, genetics, statistical framework

Personalized nutrition is an approach that is currently attracting increasing interest in the area of sport and exercise. The prospect of individually tailored, and therefore more effective, sport and exercise nutrition sounds appealing, but research in this area is still in its infancy. To compliment traditional analysis of group mean response, studies are trying to describe the totality of responses by providing individual performance and health data to an intervention which often show large inter-individual variability. Emerging evidence indicates that sport nutrition strategies may work in some individuals or under certain conditions, yet not in others, likely due to a myriad of environmental and genetic factors, highlighting the necessity in providing a more thorough examination of results. The current Research Topic aimed to provide a platform for original data and reviews on novel strategies for personalized sport and exercise nutrition.

The first original article in this Research Topic investigated whether a personalized hydration strategy based upon sweat rate influenced cardiovascular and thermoregulatory responses and exercise capacity in the heat (de Melo-Marins et al.). Participants performed a time-to-exhaustion cycling protocol at 70% of previously determined maximal workload (~38 min) on three occasions during which they consumed water according to their sweat rate, *ad libitum*, or no fluid. Although the personalized hydration strategy avoided dehydration resulting in lower skin temperature increases and end heart rate, exercise capacity was not different between sessions. This suggests that although an individualized hydration strategy may attenuate thermal strain during exercise in the heat, this does not necessarily translate into performance improvements. Wardenaar et al. used a novel method to monitor the dietary intake of 5 male ultramarathon runners during a 120 km race; researchers used bicycles to follow and film the competitors throughout the race. Large variation in carbohydrate intake was shown between runners, and consumption was well below the 90 g·h^−1^ recommendation for exercise of this duration. Furthermore, only one runner avoided dehydration >2% suggesting that recommendations should be individualized to optimize personal intakes for future races.

Two studies focussed on individual responses to ergogenic aids. Stautemas et al. investigated the pharmacokinetics of beta-alanine in blood following beta-alanine supplementation individualized according to the anthropometric parameters of each participant (10 mg·kg^−1^ body weight) and compared it to a fixed dose of 1,400 mg. Body weight was correlated to the pharmacokinetics and explained a large part of the variation in responses, although the dose individualized to body weight did not reduce this variation in beta-alanine plasma responses. These data demonstrate that there is a heterogenic response to supplementation in an anthropometrically diverse sample suggesting further research is warranted to optimize beta-alanine supplementation. Another study showed that acute caffeine (6 mg·kg^−1^ BM) intake increases time-to-exhaustion during a supramaximal effort without any change in estimated anaerobic energy contribution (Miyagi et al.). Individual analysis of the data showed that 10 out of the 14 participants increased exercise capacity above the smallest worthwhile change. Similarly, anaerobic capacity was modified beyond the limits of the smallest worthwhile change in 11 individuals, although the direction of change was highly variable; 4 increased their anaerobic contribution while 7 showed a reduction. Taken together, these data highlight the individual nature of responses to ergogenic supplements and provides scope for further work to elucidate the modifying factors behind these differences.

Two review articles were published in this Research Topic, focusing on the optimization of supplementation with extracellular buffering agents (Heibel et al.) and personalization of nutrition for athletic performance based upon genetics (Guest et al.). Heibel et al. highlighted several factors which may modify the ergogenic effects of buffering supplements such as sodium bicarbonate, sodium citrate and calcium and sodium lactate. These include the timing and dose of supplementation, factors relating to the exercise activity undertaken (e.g., intensity and duration), genetic factors, training status of the individual, and uncomfortable side-effects. The review of Guest et al. outlined a number of genetic factors that may influence absorption, metabolism, uptake, utilization and excretion of nutrients, and food bioactives, which may contribute to physical performance. Specifically, they highlight the current lack of randomized, controlled trials examining the effects of genetic variation on exercise performance in response to nutrients and other food components which may direct future nutritional prescription for athletic performance.

Finally, Swinton et al. provided a statistical framework to support researchers and applied practitioners alike who wish to determine individual responses to a nutritional intervention. This narrative review provides an overview of the fundamental concepts of measurement error and how typical error and confidence intervals can be used to determine uncertainty in measurements. Guidance on how to assess whether meaningful changes have occurred following an intervention is then provided, before discussing the challenges in identifying response or non-response. A modifiable automated spreadsheet is provided free to download to incorporate personal data sets and readers can follow the procedures described within the review to analyse their own responses. Importantly, this statistical framework does not have to be limited to nutritional interventions and can be applied to any dataset wishing to determine an individual's response.

This Research Topic has resulted in some novel articles that have furthered our knowledge in the area of personalized sport and exercise nutrition. We hope that this topic will act as a potent stimulus for further research in this exciting area.

## Author Contributions

BS, AE-S, and WD are responsible for the writing of the manuscript. All authors approved the final version of the manuscript.

### Conflict of Interest Statement

The authors declare that the research was conducted in the absence of any commercial or financial relationships that could be construed as a potential conflict of interest.

